# Progress in Medicine

**Published:** 1896-10-03

**Authors:** 


### Progress in Medicine.

### RENAL DISEASES.

**Pyelonephritis and Tuberculosis.**—Bazy[14] thinks that the proof that infection ascends from the bladder by the ureters is insufficient, and holds rather that the kidney is more often infected through the blood. Calculi, or pressure on the ureters, or a kink in them, by causing stagnation, favour the foundation of the disease. Occasionally there is spontaneous recovery, more often injection of nitrate of silver or operative measures are needed. Wunscheim[15] finds that the common cause is B. coli, less often the pyogenetic cocci, or proteus vulgaris. The pus producing microbes cause severe cases in which pyæmia nearly always occurs. Meyer[16] notices that primary tubercle of the kidney is always unilateral and local at first. Hence the importance of early surgical interference. When bacilli cannot be found, the cystoscope shows the mouth of one ureter congested with patches of inflamed mucous membrane near it. The diagnosis of these diseases from intermittent hydronephrosis may be difficult, as in a case of Tuffier's.[17] Severe attacks of pseudorenal colic followed by copious micturition without any blood occurred from time to time. There was a little pus constantly present in the urine at all times, some swelling in the right lumbar region, especially before an attack, but never any fever. The latter may be absent, indeed, in true pyonephritis, but the urine here became quite clear soon after the attacks, and was perfectly normal except for the few pus cells, which turned out to be vesical in origin and due to catheterisation. Tuffier diagnosed hydronephrosis, and this proved correct. Mabel Blackwood[18] recently showed two cases of congenital hydronephrosis with sacculated ureters dilated to the size of the large intestine in parts. There may be a variable tumour in the lumbar region, and a suddenly increased flow of urine at times. When the disease is on one side only, there may be little inconvenience in favourable cases. If we have pus and albumen both present in urine, it is desirable to estimate the albumen which is not due to the pus. K. Reinecke[19] counts the pus cells like ordinary blood corpuscles, on a slide. About a hundred thousand pus cells in the cubic centimetre cause one per cent. of albumen. The urine must be well mixed, and mucus or ammoniacal urine may lead to errors.

**Albuminuria.**—Harris[20] has devised a test of extreme delicacy for albumose. He finds that true peptone hardly ever occurs; that the albumoses are formed by the digestive action of microbes on the albumens of the body, and are thus indicative of infective action or suppuration. They are produced in excess, absorbed by the blood, and excreted by the kidneys. He claims to have diagnosed the existence of pus by means of the presence of these albumoses. To discover them he boils the urine with lead chloride and salicyl-sulphonic acid, filters, cools, and precipitates the excess of lead with sulphate of soda. The filtrate is treated with a few drops of salicyl-sulpho-tungstate of sodium, which causes a cloud with albumoses or peptone. This disappears on boiling, and the test is so delicate that it will show one part in 50,000. Lockhart Gillespie[21] quotes Osler as doubting the clinical importance of albumosuria, and points out that a part of the albumen excreted by the kidney may be changed into an albumose in the urinary tract, or on standing, though not to any large extent. He reports two cases of gouty origin with little or no albumen and no tube casts, but both presented albumoses. Here there was no pus or infectious disorder, and he suggests that, as according to Horbaczewsky there would be considerable leucocytosis, the breaking up of these corpuscles might bring about the presence of albumoses. As the clinical symptoms of these patients were grave it remains to inquire whether albumoses may not be symptomatic of lesions as dangerous in certain cases as those shown by a quantity of albumen. Various views have been taken of the effects of ether and chloroform on the kidney. Friedlander[22] found that 16 per cent. of his cases showed no albumen after the use of chloroform, thirty-seven a transient albuminuria, and in forty-seven who had albumen previously it was nearly as often increased as diminished. A writer in the *Therapeutic Gazette*[23] finds that the most recent investigations have gone to prove the harmlessness of anæsthesia. Thus Weir reported that ether at the most caused only a slight, temporary albuminuria; Feuter noticed it but rarely, Buller once in five hundred cases, Korte in one per cent., Selbach even denied that repeated etherization ever produced nephritis in animals. Kant and Mester, indeed, after prolonged chloroform narcosis, met with an unknown sulphur compound, and Becker discovered that a temporary acetonuria usually follows anæsthesia, and in diabetics this adds to the risk. Barbacci and Bebi,[24] however, found albumen in 29 per cent. after ether, and 18 per cent. after chloroform, and while the kidney lesions were temporary after ether, they were of a more permanent type when caused by chloroform. Eisendrath[25] noticed albumen to the extent of 25 per cent. after ether, and 32 per cent. after chloroform in patients previously free from it. These different results partake of the uncertanty of most statistics on anæsthesia, but newer and more perfect methods have at least not shown any increase in the albuminuria which has long been known to occur in some cases.

Among new tests for albumen we notice Barral's Aseptol, which is highly sensitive but precipitates mucus and peptones;[26] also Yavorovsky's, which contains one part of molybdate of sodium, four of citric acid, and forty of water. A drop added to filtered urine with an excess of carbonate of soda gives a cloud in the presence of albumen. Pierre Maril, in discussing a case of cyclic albuminuria,[27] takes the view that his patient's affection was a nervous one, dependent on irritation of the sympathetic. It had existed at least seven years, disappeared when lying in bed, and was accompanied with peculiar sensations in the lumbar region before a thunderstorm, and the patient suffered at times from hemidriosis. It was thus analogous to a migraine rather than to nephritis. Albuminuria in syphilis is, according to Heller,[28] very rare in the eruptive stage, but he considers that it often arises from the mercury employed in the treatment. He examined over 300 patients in a syphilis hospital and found that it generally appeared on the twelfth day of mercurial treatment, more often after inunction than after injections, where the amount used was less. Neither treatment was ever commenced until secondary symptoms were seen, and the albuminuria, indeed, was neither severe or common. Walti[29] shows that atropine diminishes the secretion of urine independently of the pressure or altered metabolism. However, his experiments were made on rabbits with larger doses than could be given to men. Alcaptonuria[30] has been noted in a fatal case of tuberculosis. The urine became dark on exposure to air or when alkalies were added, contained much indican, and reduced copper salts. It has only been seen hitherto as a chronic disease. The presence of casts in the urine without albumen is shown by the centrifugal machine to be far more frequent than was supposed. Kossler[31] found them in 29 cases of fever and other disorders. Though numerous and of all kinds he does not attach much importance to them, and in some instances, from these very cases, the kidneys appeared normal after death.

**Treatment.**—Friedrich[32] confirms the statement that great diuresis can be produced by the administration of urea. Where the kidneys were intact seven times the previous amount was excreted in a case of cirrhosis of the liver. Diuretin is reported upon by L. Vintras.[33] He finds that where the kidney disease is primary and the parenchyma much affected, or where much albumen exists, it has little value, but where the disease is secondary and only the epithelium of the tubules affected it is of great utility.

C. J. Proben[34] discusses the treatment of uræmia by pilocarpine, and concludes against it as far more dangerous and inefficient than rectal or venous injections of saline fluid, to which may be added venesection. Blake White speaks more favourably of pilocarpine if given in small doses and guarded by strychnia or digitalis, provided the heart is healthy. Lactate of strontium was found by Bronovski[35] to cause great diuresis in various forms of nephritis, but the albumen was not affected, nor was there much increase in the solids excreted. It is clear that it did not act by preventing intestinal sepsis, for the aromatic sulphates were not diminished. General treatment in France of acute nephritis is described by Lyonnais[36] as commencing with mustard poultices, or dry cupping and milk diet, to which may gradually be added green vegetables, fats, and starches. Vapour baths, and especially pilocarpine, are employed with great care. Purgatives, oxygen inhalations, and in later stages the iodides, are also found often of value. With regard to vegetarian diet an editorial in the *British Medical Journal*,[37] while speaking of its value in acute or progressive forms of the disease or where uræmia is impending, is in favour of moderate mixed diet, with a little meat in chronic cases. However, soups, beef-tea, and meat extracts are highly injurious at any stage. Huchard[38] discusses three types of dyspnœa, distinct from true uræmia, which he calls toxic, as caused by ptomaines. These are (*a*) renal, with high tension and slight albuminuria; (*b*) cardiac in cardiac sclerosis, arhythmia, &c.; (*c*) aortic, with various aortic lesions. He regards them as curable, and is wont to prescribe a pure milk diet for fifteen days; then a mixed diet for five days, with a very little meat and much milk and vegetables. He forbids all but the plainest food, and gives theobromine for several days during the fortnight of milk diet, and sodium iodide during the mixed diet. This regimen must be continued for some time. W. H. McEnsoe[39] confesses that the value of the action of the skin is always small, and especially when it has been dry for some time. Hence it should be oiled before applying hot packs, and diaphoresis should not be attempted when a cathartic is being used. He recommends for chronic renal sclerosis pills containing corrosive sublimate, digitalis, squills, and quinine. (Quinine has sometimes been said to be incompatible with the sublimate.) If the tension and arterial sclerosis are marked he gives sublimate with iron and nitrous ether or nitro-glycerine, as well as intestinal antiseptics. When the pulse is hard and rapid he advises venesection or veratrum viride. Barré[40] advocates in uræmia and infectious diseases a combined bleeding and the intravenous injection of salines. This method is only to be used when ordinary remedies fail. Two needles with tubes attached are inserted, one into a vein of each arm. Blood flows out by one, and saline fluid to an equal quantity enters by the other. An amount of 500 to 1,000 grammes can thus be replaced without affecting the existing blood pressure in 30 to 50 minutes. Toxines are removed and those left are diluted, the severe symptoms disappear, and the patient soon falls asleep in comfort. Barré thus saved three patients who were practically moribund, but who made good progress after the operation, which he calls "disintoxication of the blood."

[14] Med. Week, Ap. 3. [15] B. M. J., Ap. 18. [16] Med. Chronic., July. [17] Med. Week, May 1. [18] Edinb. Med. J., April. [19] Internat. Med. Mag., March. [20] Amer. J. Med Sciences, May. [21] Lancet, July 11, [22] B. M. J., July 27. [23] Therap. Gazette, May 15. [24] B. M. J., July 18. [25] Amer. J. Med. Sciences. [26] B. M. J., Ap. 11. [27] Med. Week., June 5. [28] Practit., April. [29] Med. Chron., March. [30] B. M. J., March 7. [31] Am. J. Med. Sciences. Ap. [32] B. M. J., July 27. [33] Lancet, Ap. 25. [34] Med. Rec., June 13. [35] Les Nouv. Remédes, Mar. 24. [36] Med. Rec., May 23. [37] B. M. J., July 25. [38] Amer. J. Med. Sciences, April. [39] Med. Rec., June 27. [40] Med. Press, June 10.

### DIABETES.

**Etiology and Pathology.**—James[1] draws attention to some interesting points in the affection. Of twelve cases in his wards, heredity could be elicited in two only, and these were both patients below eighteen years of age. He considers alcohol is often a factor in its causation, for alcohol, like morphia or chloroform, is very apt to produce glycosuria. We know that drinking bouts are often accompanied by temporary glycosuria, and if drinking bouts become common we can easily understand that the glycosuria may become permanent. Referring to a particular case, he remarks that hard work, exposure, and alcohol had all had their share in inducing his disease. He further directs attention to the condition of the blood. In the patient referred to, the number of red corpuscles was 6,100,000, and the hæmoglobin 106 per cent. Now, this extra richness in corpuscles and hæmoglobin, which is frequent in this disease, is explained by many as being due to a concentration or a thickening of the blood, the result of polyuria. But, were this the case, the specific gravity of the blood would be correspondingly increased; and, further, we should find that the specific gravity, the number of corpuscles, and the percentage of hæmoglobin should vary greatly with the amount of food, and especially of water, ingested. In two cases James found the results of his examination might be briefly summarised thus:—

Case. Between Five and Six a.m.

L.—Corpuscles, 6,232,000. Hæmoglobin, 110 p.c.; sp. gr., 1,058.

A. " 6,316,000. " 100 " " 1,053.

Case. Between Ten and Eleven a.m.

L.—Corpuscles, 6,100,000. Hæmoglobin, 106 p.c.; sp. gr., 1,0[illegible]

A. " 5,056,000. " 100 " " 1,050.

These results show that, after digestion of food and a large quantity of water, a slight diminution alike in the specific gravity, corpuscles, and hæmoglobin is remarked; but the fact which stands out prominently is that, without any marked alteration from the normal specific gravity of the blood, the proportion of corpuscles and of hæmoglobin remains specially high. James is, therefore, in the habit of ascribing this characteristic of the blood in diabetes again to the "*vis mediatrix naturæ.*" The amount of oxidisable materia- being lessened by the enormous loss of sugar in the urine, Nature tends to compensate for this by increasing the oxidising elements in the blood, viz., the corpuscles and hæmoglobin. Something analagous seems to occur in other conditions, notably in starvation. If an animal be completely deprived of food, the nutrition of the more important tissues of the nervous system and the heart is obtained by the using up of the less important, *e.g.*, the fat, muscles, glands, &c.; and, with a rapid diminution of the body weight as a whole, the blood seems to show little diminution either as regards corpuscles or hæmoglobin. It must not be forgotten, however, that in certain cases of diabetes a lessening in the richness of the blood will be found. But James believes that he is correct in saying that in the earlier cases, and in cases which have still some vigour left, it will be found the rule to meet with no diminution, but even a distinct increase in this respect.

Diabetes in early life is usually a very grave and progressive disease, but it is not so uniformly progressive as we are generally led to believe. A case in point is recorded by Edgar Duke[2], that of a girl, aged eighteen, in whom glycosuria was detected at the age of thirteen. By treatment and diet the disease had been controlled for five years. During the time she had grown from a delicate-looking child into a rather more robust young lady. Though sugar had been frequently found in the urine, long intervals had occurred during which none had been detected. The elimination of sugar was controllable by diet, and by keeping down the carbohydrates in the food, administering aperients when necessary, maintaining the condition of the blood by iron, and, as direct controllers of the sugar, sulphonal and afterwards codeia, the disease had been controlled. After extirpation of the pancreas in the dog, Kaufman[3] finds that there is inevitably glycosuria, whether the animal is fasting or digestion is in progress. Food increases glycosuria, but even prolonged fasting does not entirely arrest it. Under ordinary conditions, glycosuria, confined merely to the digestive period, is only observed when extirpation of the pancreas is incomplete. In a febrile condition, indicated by a considerable rise in the rectal temperature, the sugar disappears from the urine of diabetic animals deprived of food, but reappears when the fever ceases. Further, Kaufmann has found invariably that in operating on dogs of the same age, weight, and degree of fatness, under exactly the same conditions, during inanition the diabetic animals lose weight much more rapidly than the normal animals, the latter losing from 160 to 175 grammes to a loss of 250 to 500 grammes daily in the former. Thus denutrition is emphasised in diabetic subjects.

The cutaneous manifestations are often attributed by the patient and medical attendant to almost every possible cause but the correct one, and glycosuria is not infrequently overlooked as the real determining cause in a number of cases. Prince Morrow[4] divides the skin affections of diabetes into two classes; the first class comprising affections which are impressed with certain special characters, which reveal their diabetic origin and nature; they are sufficiently common to entitle them to a definite place in the symptomatology of diabetes. Of these he mentions certain functional and structural lesions of the skin, pruritus, diabetic erythema, eczema, balano posthitis, phimosis, furuncles, carbuncles, and gangrene, to which may be added a comparatively recently recognised dermatosis, xanthoma diabeticorum. In the second class Morrow places affections which occur incidentally and with comparative infrequency, though their association with diabetes cannot be regarded as a coincidence merely; they exhibit certain peculiarities in their clinical behaviour, especially in their tendency to recovery or retrogression, according to the fluctuation in the amount of sugar present. Among the most important may be mentioned acne cachecticorum, chronic papular urticaria, impetiginous and lichenoid eruptions, psoriasis, dermatitis herpetiformis, herpes zoster, malperforant, erysipelas, and dermatitis diabetica papillomatosa. So common and characteristic are the affections of the first class that they are recognised by French authors as a separate group, under the designation of *diabétides*, on the ground that they bear the same relation to the constitutional disorder as do the syphilides and scrofulides to their respective diatheses.

Regarding xanthoma diabeticorum, it is, as its name implies, an affection peculiar to diabetes, Morrow states that of the twenty-one cases reported (eleven of which have been recorded within the past five years), glycosuria was traced as the etiological factor in all but three. Its casual connection is further shown by the involution of the eruption, when the sugar is suppressed, and its redevelopment with the increase of the sugar. The eruption consists of small pea-sized tumours of a rounded or conical form, discrete or confluent, some of them surrounded by a yellowish apex, which gives them a deceptive resemblance to pustules; they are of solid consistence, and firm to the feel. They are essentially inflammatory and subjective sensations of heat, burning, and itching are almost invariably present. Their most common seats are the forearms, buttocks and knees, from which they may gradually extend over the extensor surfaces, the scalp, face, trunk, and abdomen; in some cases the rash is universal. The eruption may remain stationary for months or years, and then undergo rapid involution, leaving no trace; but it may redevelop two or three times. Continuing, Morrow remarks that this affection is differentiated from xanthoma by the rapidity of its development, the character of the papules, which are hard, firm, and inflammatory; by their seats of predilection, spanning the eyelids; by its invariable presence in the form of circumscribed papules, never occurring in striæ or plaques; by the subjective sensations; by the facility of its retrogression, which may be spontaneous, or the result of the correction of the glycosuria.

Triboulet[5] discusses the hepatic lesions of diabetes, and refers to the following pathological changes which have been recorded: Hypertrophy, owing to hyperæmia, to interstitial hepatitis, or to proliferation of the hepatic cells, fatty degeneration, and atrophy. The more recent researches of Hanot have revealed the existence of a portobiliary cirrhosis, with endophlebitis, in which it is possible that sugar in excess plays the same rôle as alcohol in the perilobular venous cirrhosis.

In addition to these changes must be added a number of cases which have been recorded of pigmentary hypertrophic cirrhosis, and bronzed diabetes (Hanot et Chauffard, Letulle, Brault et Galliard, Barth, Marie, &c.), Glénard has found hepatic changes in 60 per cent. of diabetic patients; hypertrophy in 35 per cent. of the cases.

[1] Ed. Med. Journ., April, 1896. [2] Birm. Med. Rec., No. 3, 1896. [3] Med. Week, March, 1896. [4] Med. Rec., Vol. xlix., No. 15. 1896. [5] Gaz. hebdan de Méd. et de Chir., No. 16, 1896; Med. Chron., June, 1896.

